# Ibrutinib continues to influence the therapeutic landscape of chronic lymphocytic leukemia: new data presented at ASCO 2017

**DOI:** 10.1186/s12916-017-0920-7

**Published:** 2017-08-16

**Authors:** Stefano Molica

**Affiliations:** Department Hematology-Oncology, Azienda Ospedaliera Pugliese-Ciaccio, Viale Pio X, 88100 Catanzaro, Italy

**Keywords:** Ibrutinib, Novel association, ASCO 2017, Chronic lymphocytic leukemia

## Abstract

According to data presented at the 2017 American Society of Oncology (ASCO) Annual Meeting, with more than 4 years of follow-up, ibrutinib continues to provide clinical utility in chronic lymphocytic leukemia (CLL). However, treatment of CLL patients with high-risk cytogenetics features remains a challenge and the outcome of these hard-to-treat patients is dismal. At the 2017 ASCO Meeting, results of the GENUINE phase III trial showed that, by adding ublituximab, a glycoengineered, anti-CD20 type 1 monoclonal antibody, to ibrutinib, the overall response rate (ORR), complete response rate (CRR), and minimal residual disease (MRD) negativity may be improved in high-risk CLL patients. A further way to improve the results obtained with Bruton’s tyrosine kinase (BTK) inhibitors is the parallel use of ibrutinib with chimeric antigen receptor (CAR) T-cell therapy. Through this investigational approach, the rate of MRD negativity was shown to be higher, implying potential eradication of CLL. These novel data indicate that ibrutinib continues to have a positive effect in CLL.

## Background

With the regulatory approval of ibrutinib, idelalisib, and venetoclax, as well as of other therapeutic small molecules likely to become widely available in the coming years, the treatment of chronic lymphocytic leukemia (CLL) has changed dramatically. However, complete remissions (CRs) are rare in CLL and treatment options for patients relapsing after treatment with ibrutinib remain limited [1]. The potential synergy of ibrutinib with other treatment strategies, including immunotherapeutic and targeted approaches, is currently being investigated in various clinical trials with the hope to improve either the depth or duration of response.

At the 2017 American Society of Oncology (ASCO) Annual Meeting, investigators presented mature results from key ibrutinib clinical trials and emerging data on novel associations with ibrutinib, demonstrating activity against highly resistant, harder-to-treat CLL. Between the new drugs (i.e., ibrutinib, idelalisib, and venetoclax) and immunotherapeutic approaches, such as chimeric antigen receptor T-cell (CAR T-cell) therapy, there is great hope for the future treatment of CLL patients.

## The RESONATE trial

Pivotal RESONATE data on ibrutinib, a first-in-class Bruton’s tyrosine kinase (BTK) inhibitor, have significantly transformed the treatment landscape for patients with relapsed or refractory CLL [2]. An update of the RESONATE trial, presented at the 2017 ASCO Meeting, continues into the fourth year of study to show a favorable impact of ibrutinib on survival outcomes in relapsed or refractory CLL patients [3]. With a median follow-up time of 44 months, progression-free survival (PFS) was still significantly longer for ibrutinib than ofatumumab (median not reached versus 8 months; hazard ratio [HR] 0.133; *P* < 0.0001; 3-year PFS 59% versus 3%). The significant benefit was observed across patient subgroups with genomic abnormalities usually considered at higher risk of progression. Patients with deletion 11q tended to have the most favorable outcome; furthermore, PFS was not statistically different for patients with deletion 17p or deletion 11q or without these FISH abnormalities. The safety profile of ibrutinib was consistent with previous reports [1]. Major hemorrhage, Common Terminology Criteria for Adverse Events (CTCAE) grade 3 atrial fibrillation, or CTCAE grade 3 hypertension occurred in 6–8% of patients. Interestingly, the incidence of most grade 3 adverse events decreased over time. Discontinuations were more frequently due to progression of CLL (27%) and adverse events (12%). At the time of analysis, 90 (46%) study patients continued on ibrutinib [3].

## Optimal sequencing of kinase inhibitors (KIs) in CLL

Although results of the RESONATE trial highlight the value of ibrutinib in the treatment of CLL, to date, hematologists have little guidance on which kinase inhibitor (KI) (i.e., ibrutinib or idelalisib) should be used first [4]. A recent study by nine large US cancer centers and the Connect CLL Registry provides further indication on this regard [5]. In this retrospective study that analyzed records of 683 CLL patients treated with KIs (i.e., 621 received ibrutinib and 62 received idelalisib), researchers looked at patient demographics, discontinuation rates and reasons, overall response rates, survival, and post KI salvage strategies. Interestingly, patients treated with ibrutinib experienced a PFS nearly three times longer than patients who received idelalisib, both as first-line therapy and for relapsed/refractory CLL. Authors concluded that, in the largest experience of novel agents published to date in CLL, ibrutinib appears superior to idelalisib in all settings as a first choice KI [6].

## Combining ibrutinib with immunotherapy – The GENUINE Trial

Ibrutinib has been associated with an anti-CD20 monoclonal antibody under the assumption that the latter would help reduce the lymphocytosis often observed with ibrutinib single agent [6]. Rituximab, in combination with ibrutinib, has been the most widely used anti-CD20 monoclonal antibody in CLL [7]. The approach makes sense, as CLL cells vacate the lymph node and bone marrow environments in which they thrive and become vulnerable in blood.

Ublituximab is a glycoengineered, anti-CD20 type 1 monoclonal antibody that maintains complement-dependent cytotoxicity and enhances antibody-dependent cell-mediated cytotoxicity [8]. At the 2017 ASCO Meeting, Sharman et al. [9] reported results of a phase 3 study (the GENUINE trial) aimed at investigating whether the addition of ublituximab to ibrutinib would improve the outcome of patients with relapsed/refractory genetically high-risk CLL. The ibrutinib dose was 420 mg daily in both arms, and the ublituximab dose was 900 mg on days 1, 8, and 15 of cycle 1, followed by dosing on day 1 of cycles 2 through 6.

In the combination arm, the best overall response rate was 78%, with 7% achieving CR, compared with 45% in the monotherapy arm with no CRs (*P* < 0.001). PFS showed a trend toward improvement in the patients treated with the combination, with a HR of 0.559, albeit not statistically significant at the time of analysis. The investigators concluded that the study met its primary endpoint, with a superior and deeper response rate of association in comparison to ibrutinib single agent; however, the results should be interpreted with caution due to the absence of a PFS improvement [9]. To date, the association of ibrutinib and an anti-CD20 monoclonal antibody cannot be recommended over ibrutinib single agent in daily practice.

## Introducing CAR T-cell therapy into conventional CLL treatment

A challenging issue is that of introducing immunotherapy with anti-CD19 CAR T-cell therapy into conventional CLL treatment [10]. At the ASCO 2017 Meeting, Gill et al. [11] reported on the findings of a pilot trial assessing the association between ibrutinib and CAR-T cell therapy, including 10 CLL patients who had failed at least one regimen before ibrutinib, unless they had 17p deletion or TP53 mutation. After a median follow-up of 6 months, eight of the nine evaluable patients achieved minimal residual disease (MRD) negativity in flow cytometry and all remained with bone marrow CR at last follow-up. Cytokine release syndrome developed in nine patients and was grade 1 in two patients, grade 2 in six patients, and grade 3 in one patient. Importantly, treatment of cytokine release syndrome with the IL-6 receptor antagonist tocilizumab was not required [11]. In this pilot trial, the attainment of MRD negativity opens up future discussion on the possibility of stopping ibrutinib therapy to obviate the need for chronic therapy. However, an unaddressed issue is how results of this study translate into more clinically relevant endpoints such as PFS and overall survival.

## Conclusions

In conclusion, the ASCO 2017 Annual Meeting showcased a number of important studies expected to influence the scenario of CLL treatment in the near future. In particular, strategies of ibrutinib association are likely to contribute to the complete eradication of disease from the bone marrow and to potentially cure CLL.

## References

[1] Molica S (2017). Targeted therapy in the treatment of chronic lymphocytic leukemia: facts, shortcomings and hopes for the future. Expert Rev Hematol.

[2] Byrd JC, Brown JR, O'Brien S (2014). Ibrutinib versus ofatumumab in previously treated chronic lymphoid leukemia. N Engl J Med.

[3] Byrd JC, Hillmen P, O'Brien SM, et al. Long-term efficacy and safety with ibrutinib (ibr) in previously treated chronic lymphocytic leukemia (CLL): Up to four years follow-up of the RESONATE study. J Clin Oncol. 2017;35:(suppl; abstr 7510)

[4] Zelenetz AD (2017). Chronic lymphocytic leukemia: individualizing treatment approach. J Natl Compr Canc Netw.

[5] Mato AR, Hill BT, Lamanna N (2017). Optimal sequencing of ibrutinib, idelalisib, and venetoclax in chronic lymphocytic leukemia: results from a multicenter study of 683 patients. Ann Oncol.

[6] Burger JA, Keating MJ, Wierda WG (2014). Safety and activity of ibrutinib plus rituximab for patients with high-risk chronic lymphocytic leukaemia: a single-arm, phase 2 study. Lancet Oncol.

[7] Jain P, Keating MJ, Wierda WG (2017). Long-term follow-up of treatment with ibrutinib and rituximab in patients with high-risk chronic lymphocytic leukemia. Clin Cancer Res.

[8] Sharman JP, Farber CM, Mahadevan D (2017). Ublituximab (TG-1101), a novel glycoengineered anti-CD20 antibody, in combination with ibrutinib is safe and highly active in patients with relapsed and/or refractory chronic lymphocytic leukaemia: results of a phase 2 trial. Br J Haematol.

[9] Sharman JP, Brander DM, Mato AR, et al. Ublituximab and ibrutinib for previously treated genetically high-risk chronic lymphocytic leukemia: Results of the GENUINE phase 3 study. J Clin Oncol. 2017;35:(suppl; abstr 7504).

[10] Fraietta JA, Schwab RD, Maus MV (2016). Improving therapy of chronic lymphocytic leukemia with chimeric antigen receptor T cells. Semin Oncol.

[11] Gill S, Frey NV, Hexner EO, et al. CD19 CAR-T cells combined with ibrutinib to induce complete remission in CLL. J Clin Oncol. 2017;35:(suppl; abstr 7509).

